# Immunogenicity assessment strategy for a chemically modified therapeutic protein in clinical development

**DOI:** 10.3389/fimmu.2024.1438251

**Published:** 2024-11-11

**Authors:** Charlotte Hagman, Gaetan Chasseigne, Robert Nelson, Florian Anlauff, Mark Kagan, Allison B. Goldfine, Grzegorz Terszowski, Maria Jadhav

**Affiliations:** ^1^ Pharmacokinetic Sciences - Drug Disposition, Biomedical Research, Novartis, Basel, Switzerland; ^2^ BioAgilytix Laboratories, Hamburg, Germany; ^3^ Pharmacokinetic Sciences, Biomedical Research, Novartis, East Hanover, NJ, United States; ^4^ Translational Medicine, Biomedical Research, Novartis, Cambridge, MA, United States; ^5^ Pharmacokinetic Sciences - Drug Disposition, Biomedical Research, Novartis, Cambridge, MA, United States

**Keywords:** immunogenicity, clinical development, therapeutic proteins, chemical modification, endogenous counterpart

## Abstract

The clinical immunogenicity assessment for complex multidomain biological drugs is challenging due to multiple factors that must be taken into consideration. Here, we describe a strategy to overcome multiple bioanalytical challenges in order to assess anti-drug antibodies (ADA) for a novel and unique chemically modified protein therapeutic. A risk-centered approach was adopted to evaluate the immunogenic response to a modified version of human growth differentiation factor 15 (GDF15) connected to an albumin-binding fatty acid via a polyethylene glycol (PEG) linker. Key steps include monitoring anti-drug antibodies (ADAs), using a standard tiered approach of screening and confirmation. To deepen our understanding of ADA response, as a third tier of immunogenicity assessment, novel extensive characterization using a set of assays was developed, validated, and used routinely in clinical sample analysis. This characterization step included performance of titration, mapping of ADA response including anti-GDF15 and anti-PEG–fatty-acid antibody characterization, and assessment of the neutralizing anti-drug antibodies (NAbs) using cell-based assays for immunogenicity in parallel. The analytical methods were applied during two clinical trials involving both healthy volunteers and overweight or obese patients. We observed low incident rates for ADA and no ADAs against the PEG linker with fatty acid conjugation. In one of the clinical studies, we identified neutralizing ADAs. The proposed novel strategy of extensive characterization proved effective for monitoring the presence of ADAs and NAbs and can be used to support clinical development of a broad range of chemically modified proteins and multidomain biotherapeutics.

## Introduction

The first monoclonal antibody therapeutic was approved by the Food and Drug Administration (FDA) in 1986 (1), consisting of human/mouse chimeric sequences. Since this time, drug developers have worked extensively to improve the pharmacokinetic (PK), pharmacodynamic (PD), and immunogenicity profile of therapeutic antibodies and proteins primarily through sequence optimization. More recently, structural and chemical modifications such as PEGylation (2), glycosylation (3), and lipidation (4) have been introduced resulting in multidomain biotherapeutics (MDB) (5, 6) with enhancements in stability, aggregation, adsorption, and degradation, in addition to PK, PD, and immunogenicity improvements. For example, in 2013, a recombinant anti-hemophilic factor VIII was approved to treat and prevent bleeding in patients with hemophilia A (7, 8). The medicine required several doses per day during bleeding episodes. To improve the pharmacokinetic properties and reduce the patient burden of frequent administration, the active substance was chemically modified by linking a polyethylene glycol (PEG) polymer chain (PEGylation) resulting in a new active substance with reduced clearance and a longer half-life, which was approved by the FDA in 2019 (9). However, these modifications may lead to anti-drug antibody (ADA) formation not only to the protein itself but also to the newly formed potentially immunogenic epitopes at points of chemical modification or to the PEG part of the molecule (10). Considering this, the bioanalytical strategy to monitor ADAs during clinical development must address these concerns by developing multiple assays for the ADA characterization.

Immunogenicity can lead to failure of a product in the late stages of clinical development and is a critical consideration in the development of biotherapeutics. Most biologic molecules induce different levels of immune response in treated individuals, potentially leading to the formation of ADAs, the impact of which can range from no observable consequence to, in more extreme cases, substantial impacts on exposure, efficacy, and safety of the administrated drug (11). The situation becomes more complex when the drug contains homology, or partial homology, with an endogenous peptide or protein. In these cases, antibodies raised to the drug may cross-react with the endogenous counterpart, increasing the potential risk of safety-related events. Antibodies to the endogenous peptide can be sustained. Additionally, the presence and impact of preexisting antibodies is often a concern for modified or multidomain proteins, with a high prevalence reported for antibody fragments (12) and PEG (2, 10, 13).

Industry best practices (14–16) and regulatory guidance (14, 17) provide a framework to assess the incidence, magnitude, and clinical impact of the humoral (antibody) immune response to biotherapeutics. It is recommended to establish assays to monitor ADAs throughout the whole life cycle of drug development (14, 17). In the case of antibodies induced against a drug containing an endogenous counterpart, assessment of the neutralization potential of these ADAs to the drug and also to the endogenous counterpart at the entry into clinical development is often a requirement (15, 18, 19). The existence of neutralizing ADAs (NAb) is often correlated with a lower clinical response to the administered drug (20, 21).

An immunogenicity monitoring strategy was developed and implemented to support the clinical development program of a novel complex biologic therapeutic in early clinical development. For a multidomain biotherapeutics drug of this format, several aspects were considered when defining the strategy since each conjugation results in unique domain interfaces (22, 23). The risk assessment is based on numerous factors such as B lymphocyte and T lymphocyte cell epitopes, the presence of endogenous counterparts, and the formulation and availability of pharmacodynamic biomarkers (15, 17).

At the time of the initiation of the first in human (FIH) study for the described drug, no reliable clinical pharmacodynamic biomarkers for target engagement or response prediction had been previously identified that could serve as indicators of safety and efficacy. GDF15 is involved in energy regulation by suppressing food intake and is thought to play an important role in metabolic disease (24); thus, a conservative immunogenicity approach was developed. From an immunogenicity risk assessment standpoint, we assessed the drug as a high-risk molecule as it is additionally chemically modified, thereby justifying the extensive immunogenicity strategy that was proposed and implemented.

Taking into consideration the unique and novel structure of the chemically modified drug, while designing a bioanalytical strategy, we had to take into consideration both the high-risk nature of the protein part of the biotherapeutic, chemical modification that could potentially create novel immunogenic epitopes and the PEG linker. To address these considerations, a conventional assessment ADA-tiered approach with a novel extensive panel of characterization assays run in parallel was used.

The ADA assays developed for the protein therapeutic consisted of a screening assay and a confirmatory assay to detect ADA against the whole protein including modifications, and an extended set of characterization assays which were requested by regulatory authorities (14, 17). These characterization assays consisted of a titration assay to evaluate the magnitude of ADA response to the whole protein, and then two domain-specific characterization assays to assess whether ADAs were specific for endogenous GDF15 and or specific for the modification with PEG linker + fatty acid. Furthermore, two neutralizing cellular assays were developed, one for the detection of neutralizing antibodies against the whole therapeutic protein (GDF15 and PEG linker + fatty acid) and one against the protein domain of the therapeutic protein (GDF15). Together, an approach of performing these five assays applied in parallel allowed for detailed characterization of the immunogenicity response to this multidomain biotherapeutics to support a first-in-man and a proof-of-concept study.

## Materials and methods

### Source of human serum samples

Human serum samples were obtained from two clinical studies. The first was an exploratory, randomized, investigator- and subject-blinded, sponsor open-label, placebo-controlled first in-human-study of single ascending subcutaneous doses (SAD) of the therapeutic protein. The study was conducted from August 2019 through November 2021, at two research sites in the United States. The second was a non-confirmatory, randomized, placebo-controlled, participant- and investigator-blinded, sponsor open-label study in which participants received up to eight biweekly subcutaneous doses of the therapeutic protein. The study was conducted from February 2022 to May 2023, at four clinical research sites in the United States. Institutional Review Board approvals were obtained for each site for both studies from Advarra (Columbia, MD), and trials were conducted according to the Declaration of Helsinki.

### Detection of anti-drug antibodies with acid dissociation

**Figure 1:**
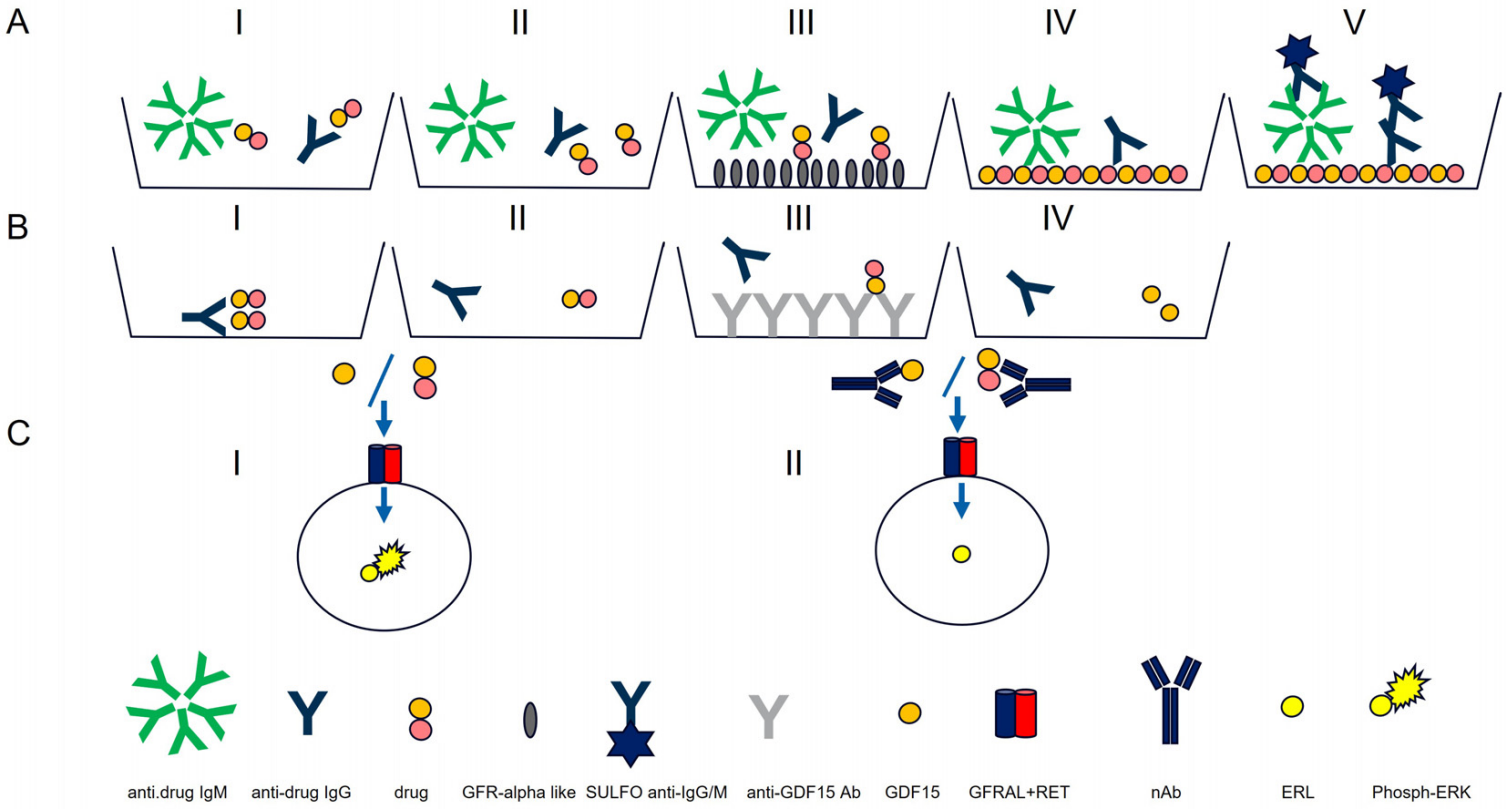
Antibody assay **(A)** and neutralizing antibody assays (Nab) **(B, C)**. Antibody assay: AI) ECL assay for IgG and IgM detection. AII) Acid dissociation to improve drug tolerance. AIII) Drug capture with immobilized GFRAL to remove access of drug. AIV) Drug-coated MSD plate used to capture IgG and IgMs. AV) Detection with SULFO-TAG polyclonal anti-human IgG and IgM. Neutralizing antibody assays: BI) PEG treatment. BII) Acid dissociation of co-precipitated drug. BIII) Drug capture by immobilized anti-GDF15 antibodies. BIV) Purified sample. Neutralizing antibody assays: CI) Purified sample mixed with GDF15 or drug and incubated with HEK293-hGFRAL/RET cells (29). Sample with no NAbs results in high ERK phosphorylation). CII) Purified sample containing NAbs mixed with GDF15 or drug and incubated with HEK293-hGFRAL/RET cells. The presence of neutralizing ADAs decreases the interaction with the hGFRAL/RET receptor resulting in a lower ERK phosphorylation.

Anti-drug antibodies (ADAs) to the chemically modified GDF15 (the drug) were detected using a validated electrochemiluminescence (ECL) assay on the Meso Scale Discovery (MSD) platform. According to the Health Authorities guidelines, the assays were developed to detect both IgG and IgM, (**Figure 1**, AI). An equimolar mix of three anti-human GDF15 monoclonal antibodies was used as a surrogate positive control antibody pool (SPC) (14).

The poor solubility of the protein therapeutic at neutral pH did not allow chemical conjugation with biotin or SULFO-TAG necessary to set up ADA bridging, and industry-standard SPEAD (Solid Phase with Extraction Acid Dissociation) and PandA (Precipitation and Acid dissociation) assay formats were implemented (25, 26). Instead, a sequential ECL immunoassay was developed with a drug-target-mediated drug removal pretreatment step to improve drug tolerance. For this purpose, complexes of anti-drug antibodies with the drug in samples were dissociated with 300 mM acetic acid (**Figure 1**, AII). Following neutralization of the acidified samples with Tris buffer, the samples were immediately transferred to nickel plates on which a His-tagged drug target (GDNF family receptor alpha-like, GFRAL) was immobilized. At neutral pH, the drug target competed with ADAs for drug binding; the drug was captured by its target and thereby removed from the samples. To reach sufficient drug tolerance, this drug removal step was repeated with a second drug-target-immobilized nickel plate (**Figure 1**, AIII). The supernatants from the nickel plates contained the un-complexed ADAs (pretreated samples). For screening and titration assays, the supernatant was then diluted with low cross buffer; for the confirmatory assays, it was diluted with the respective confirmatory agent (the drug, the GDF15 protein, or the PEG-fatty acid residue) and preincubated for 30 min before being transferred to MSD standard plates coated with the drug (**Figure 1**, AIV). Bound ADAs were then detected via their Fc region by using a combination of SULFO-TAG-conjugated goat polyclonal anti-human IgG (Southern Biotech 2049-01; Sulfo-Tag NHS Ester (MesoScale Discovery R91AO-1) and SULFO-TAG-conjugated goat anti-human IgM antibody [Southern Biotech 2020-01; Sulfo-Tag NHS Ester (MesoScale Discovery R91AO-1)] (**Figure 1**, AV). The electrical excitation of the Sulfo-Tag is mediated by a redox reaction and leads to light emission. The light intensity quantified by the system is proportional to the amount of bound antibody complexes and is output in the form of relative light units (RLUs) (**Figure 1**, I).

To distinguish between the ADA directed against the chemically modified GDF15 (the drug), the protein backbone (GDF15) and the PEG linker + fatty acid domain, three confirmatory assays were established in which an excess of chemically modified GDF15 (confirmatory I), GDF15 protein (confirmatory II), or PEG linker + fatty acid residue (confirmatory III) domain-specifically suppresses the ADA-induced signals. Validation of the confirmatory assay included definition of the confirmatory cut point (CCP) for all three confirmatory assays. Due to the exploratory nature of ADA cross-reactivity assessments in confirmatory II (GDF15) and confirmatory III (PEG-fatty acid) assays, evaluations of precision, sensitivity, and matrix effects were not conducted for these confirmatory assays, whereas the same parameters have been assessed for anti-drug confirmatory assay.

The minimum required dilution (MRD) of 1:50 in the screening and confirmatory assays represents the cumulative dilution of the samples during sample pretreatment and final dilution in LowCross-Buffer.

### Screening cut point

The screening cut point (SCP) is defined as the level of response at which a sample is screening assay positive for the presence of anti-drug antibodies. As assay responses vary between plates, a floating screening cut point is generally recommended (16, 27, 28). The requirement of a floating screening CP was shown by a positive correlation of mean sample signal level per plate versus mean negative control (NC) signal level per plate for all CP runs in scatter plot analyses. A floating screening cut point was established that uses a statistically determined screening cut point factor (SCPF) to normalize the CP to the NC of the respective plates: The plate-specific CP = SCPF × (plate mean NC). To establish the SCPF, 51 individual samples from untreated healthy subjects were analyzed in duplicate in six independent preparations in a semi-balanced design (see “Statistical Evaluation”).

### Confirmatory cut points

The CCP is defined as the inhibition percentage at or above which a sample is considered confirmed positive for anti-drug antibodies. Three CCPs were established, CCP-I, specific for the chemically modified GDF15, a characterizing CCP-II, specific for the protein backbone of the drug (GDF15), and another characterizing CCP-III, specific for the PEG linker + fatty acid moiety of the drug. To establish the CCP-I for the drug, 51 drug-naïve serum samples from untreated healthy subjects were analyzed in the presence (i.e., inhibited sample) and absence (i.e., uninhibited sample) of the drug in six independent determinations. The percentage of signal inhibition between drug-spiked and non-spiked samples is determined for each individual serum sample as follows: Signal inhibition % = [1-(signal of drug spiked sample/signal of neat sample)] * 100%. A non-parametric approach was used, with analytical outliers being removed based on Tukey box plot outlier test on stacked subject-level residuals (see “Statistical Evaluation” in “Materials and Methods”. The target false positive rate was 1% (16, 27).

To establish the CCP-II (for GDF15) and CCP-III (for PEG linker + fatty acid), respectively, 30 drug-naïve serum samples from untreated healthy subjects were analyzed in the presence (i.e., inhibited sample) and absence (i.e., uninhibited sample) of GDF15 (5 µg/mL) or PEG linker + fatty acid (1.14 µg/mL equimolar to 20 µg/mL drug) in six independent determinations. Analytical outliers were removed based on Tukey box plot outlier test on stacked subject-level residuals (see “Statistical Evaluation”). The target false positive rate was 1%.

### MBL949 and GDF15 neutralizing antibody assays

The ability of anti-drug antibodies to inhibit the drug’s or endogenous GDF-15 activity was explored in a HEK293 cell line co-expressing the human GFRAL receptor and RET receptor on the surface. pIRES plasmid including subcloned sequences encoding hGFRAL and hRET51 was used for creation of a stably transfected cell line. Alternatively, a commercially available source can be used (29). Stimulation of the HEK293-hGFRAL/RET cells with drug or GDF15 triggered an intracellular signaling cascade leading to ERK phosphorylation. ERK phosphorylation in relation to total non-phosphorylated ERK was used as assay readout. Assay controls were prepared with a surrogate positive control antibody (monoclonal antibody directed against GDF15).

Similarly to the ADA assay, the inability to chemically conjugate the drug did not allow SPEAD NAb assay formats, which would have been ideal to remove interfering matrix components such as growth factors causing unspecific ERK phosphorylation in this assay and to remove free drug to improve drug tolerance of the assay (26). Instead, all confirmed ADA-positive serum samples were pretreated by PEG treatment (12.5% PEG 6000) that precipitated unspecific antibodies, drug, and drug–ADA complexes (**Figure 1**, BI) (30, 31). To improve drug tolerance, the co-precipitated drug was released from antibody–drug complexes by 300 mM acetic acid (**Figure 1**, BII) and captured by immobilized anti-GDF15 antibodies (II) (**Figure 1**, BIII). Purified samples (**Figure 1**, BIV) were mixed with drug or GDF-15 depending on the specificity of the NAb assay (detection of neutralizing antibody assays (NAbs) against drug or against GDF15) at a concentration of 0.3 nM and added to pre-seeded HEK293-hGFRAL/RET cells. The binding of the drug or GDF15 to the GFRAL/RET complex induces ERK phosphorylation (**Figure 1**, CI). After 20 min of incubation, cells were lysed with a lysis buffer (Tris Lysis buffer, MSD R60TX-3; Protease Inhibitor Solution, MSD K15707D-3: Phosphatase Inhibitor Solution I, MSD K15707D-3; Phosphatase Inhibitor Solution I, MSD K15707D-3; AEBSF, Sigma A8456; SDS solution MSD K15707D-3), lysates were stored at −80°C till further measurement. The level of ERK phosphorylation in the cell lysates was measured as a drug/ligand activity marker using the Phospho/Total ERK1/2 Whole Cell Lysate Kit from Meso Scale Discovery. In the presence of neutralizing antibodies, drug/GDF-15 binding to the hGFRAL/RET receptor complex was inhibited, leading to a lower ERK phosphorylation level (**Figure 1**, CII). The ERK phosphorylation level inversely correlated with the amount of neutralizing antibodies present in the sample.

The ERK phosphorylation level was calculated with the phospho-ERK1/2 and total ERK1/2 signals obtained from each sample by converting them to respective % phosphoprotein values, as described in the MSD manual (Phospho/Total ERK1/2 Whole Cell Lysate Kit) using the following formula:


$$\begin{array}{c} \%\text{phospho}-\text{protein }=\;((2\;\times \text{ pERK}-\text{signal})\;/\\ \;(\text{pERK}-\text{signal }+\text{ Erk}1/2-\text{signal}))\;\times \;100 \end{array}$$

The % phospho-protein values were further normalized to the upper and lower signal controls that defined the dynamic range of the assay (upper signal controls = whole drug or GDF15 added to cells = 0% of inhibition; low signal control = cells without whole drug/GDF15 stimulation = 100% of whole drug/GDF15 inhibition).


$$\begin{array}{l} \%\text{ inhibition }=\;100-((\%\text{ phospho}-\text{protein of sample}\\ -\%\text{ phospho}-\text{protein of low signal control})/\\ (\%\text{ phospho}-\text{protein of high signal control}\\ -\%\text{ phospho}-\text{protein of low signal control}))\;*100 \end{array}$$

### Cut point determination for anti-drug/anti-GDF-15 neutralizing antibody assays

The assay cut point (CP) is defined as the level of response of the assay at and above which a sample is positive for the presence of neutralizing antibodies. Assay cut points for the anti-drug and anti-GDF15 neutralizing assays were determined by using 30 individual drug-naive self-declared healthy subjects. Those samples were analyzed in duplicate in six independent preparations (see Statistical Evaluation). To calculate the CP, the readout of each sample was normalized to internal run controls that determine the plate specific upper and lower dynamic range level (negative control without stimulating drug/negative control sample with stimulating drug) to minimize inter-run variability of the assay signal. Due to the fast ERK phosphorylation kinetics after cell stimulation, depending on the location of the sample on the plate (left third, center third, or right third), an individual normalization routine was applied to corresponding location-specific upper and lower dynamic range controls. Samples and assay performance controls located in the left third of the plate in columns 1–4 were normalized to upper and lower dynamic range controls located in the left third of the plate, whereas samples and assay performance controls located in the right third of the plate in columns 9–12 were normalized to upper and lower dynamic range controls located in the right third of the plate and samples located in the center third of the plate in columns 5–8 were normalized to the mean of the upper and lower dynamic range controls located in the left third and the right third of the plate. In total, there was one upper and one lower dynamic range control each in duplicates placed in the left third of the plate, and one upper and one lower dynamic range control each in duplicates placed in the right third of the plate. This normalization routine reduced the effect of the unpreventable plate drift effects in the raw signals on normalized signals.

Analytical outlier values in both neutralizing assays were identified using a box-plot analysis from stacked subject-level residuals (27). Subsequently, a biological outlier was identified by Tukey box plot analysis and excluded from CP determination (see “Statistical Evaluation”). The CPs for drug NAb and GDF15 NAb employed a parametric approach.

### Statistical evaluation

The statistical analysis performed to determine the ADA assay cut point was based on the strategy and considerations outlined by Shankar et al.; the concept was further developed by Devanarayan et al. Following the recommendation of using at least 50 drug-naive individual samples for cut point determination, the experimental set up of the cut point assessment was designed in a balanced fashion (27) with three sample groups (A, B, and C) comprising 17 individual samples each. Each sample group was analyzed on each of the three plates per assay run, leading to 306 data points (51 individuals, analyzed 6 times). Samples were analyzed in the screening non-drug spiked and whole-drug confirmatory in parallel.

The ADA screening cut point factor was established with mean plate NC-normalized (signal to noise) and log-transformed values with outliers being removed. Analytical outliers were identified by the evaluation of the differences of signal-to-noise results of each determination of a subject sample from the median signal-to-noise value of the corresponding subject. These obtained subject-level residuals were analyzed stacked using Tukey’s outlier box plot, where samples above and below the defined limits were considered as analytical outlier:


$$\begin{array}{c} \text{Upper outlier limit}:\;75\text{th percentile }+\;1.5\;\times \;(75\text{th percentile}\\ \;-25\text{th percentile}), \end{array}$$

$$\begin{array}{c} \text{Lower outlier limit}:\;25\text{th percentile}-1.5\;\times \;(75\text{th percentile}\\ \;-25\text{th percentile}). \end{array}$$

Acknowledging a heterogenous signal distribution in the cut point data set and the fact that excluding statistical outlier from the data set lowers the determined screening cut point factor, a conservative strategy for cut point determination was chosen with box plot constant k = 1.5 instead of k = 3, as recommended by Devanarayan et al. (27). This lowered the threshold for outlier selection and allowed the removal of more outlier values from the data set resulting in a lower screening cut point factor which enabled the detection of signal increase in low signal samples at the expense of a higher false positive rate in the screening assay.

After analytical outlier elimination (37 out of 306 data points), the medians of the remaining subject-specific signal-to-noise determinations were assessed for biological outliers using Tukey’s box plot (27). Subjects with signal-to-noise medians above or below the defined limits were considered as biological outliers and removed (three subjects with a total of eight remaining data points out of 269 data points after analytical outlier removal). The 261 remaining data points were stacked and assessed for normality. As the dataset was abnormally distributed and skewness <1, the robust parametric approach was followed. The screening cut point factor was determined as the anti-log of the median of log-transformed signal to noise values + 1.645 × (1.4826 × median absolute deviation) targeting a false positive rate of >5%.

The ADA confirmatory cut point determination for drug (CCP-I) was based on 306 data points, using the same strategy as for the screening cut point factor result. In short, % inhibition values obtained for the 51 individuals analyzed six times were assessed for analytical outlier by applying Tukey’s box plot analysis on stack subject-level residuals (individual differences of each determination to the median of the respective subject specific determinations). Analytical outliers were removed (22 out of 306 data points), and the remaining data set was assessed for biological outliers (three subjects with a total of 16 data points out of 284 data points after analytical outlier removal) by applying again Tukey’s box plot analysis on the subject specific medians. The final outlier cleaned data set (268 data points) was assessed for normality using the Shapiro–Wilk normality test, and the data set was abnormally distributed with skewness < 1. The robust-parametric method was not considered as it would result in a lower confirmatory cut point factor compared with the non-parametric approach with a confirmatory assay sensitivity < screening assay sensitivity. Considering the tired approach in which only preselected samples from the screening assay are intended for confirmatory assay analysis, for the sake of assay robustness, the higher CCP-I determined by the non-parametric approach was selected (99th percentile of all signal inhibitions excluding outliers). The CCP-I targets a false positive rate of 1%.

The ADA confirmatory cut points for the characterizing assays (GDF15 and PEG linker + fatty acid modification, CCP-II and CCP-III respectively) followed the same rationale as outlined above only that the number of drug-naive samples to be assessed six times was reduced to 30 leading to a data set with 180 data points. A parametric approach was used for CCP-II (GDF15), and a non-parametric approach was used for CCP-III (PEG linker + fatty acid), with analytical outliers (18 out of 180 data points for CCP-II, 15 out of 180 data points for CCP-III) being removed based on the box plot outlier test on stacked differences of each sample to the respective median of samples of each subject. Biological outliers (one subject with three data points out of remaining 162 data points for CCP-II after removal of analytical outlier and one subject with four data points out of remaining 165 data points for CCP-III after removal of analytical outlier) were subsequently removed based on a box plot outlier test on the respective medians of the six values for the 30 samples, leaving a final data set of 159 data points for CCP-II and 161 data points for CCP-III. CCP-II (GDF15) was calculated using the mean + 2.33* SD of all signal inhibitions excluding outliers, whereas CCP-III (PEG linker + fatty acid) was calculated using the 99th percentile of all signal inhibitions excluding outliers (27). Both CCP-II and CCP-III target a false positive rate of 1%.

For the drug and GDF15 NAb assays, 30 drug-naive samples were assessed six times (total of 180 datapoints) to establish the two cut points. The % inhibition values obtained for the 30 individuals were assessed for analytical outlier by applying Tukey’s box plot analysis on stack subject-level residuals (individual differences of each determination to the median of the respective subject-specific determinations). For the drug NAb assay cut point, 11 analytical outliers and one subject with five remaining data points as biological outliers were removed from the data set leading to 164 remaining data points. The final data set was normally distributed, and the cut point established the anti-log of the mean of % inhibition values + 2.33 × SD targeting a false positive rate of 1%. For the GDF15 NAb assay cut point, 11 analytical outliers and one subject with five remaining data points as biological outliers were removed from the data set leading to 164 remaining data points. The final data set was normally distributed, and the cut point established the anti-log of the mean of % inhibition values + 2.33 × SD targeting a false positive rate of 1% (27).

## Results

### Immunogenicity assessment strategy

**Figure 2:**
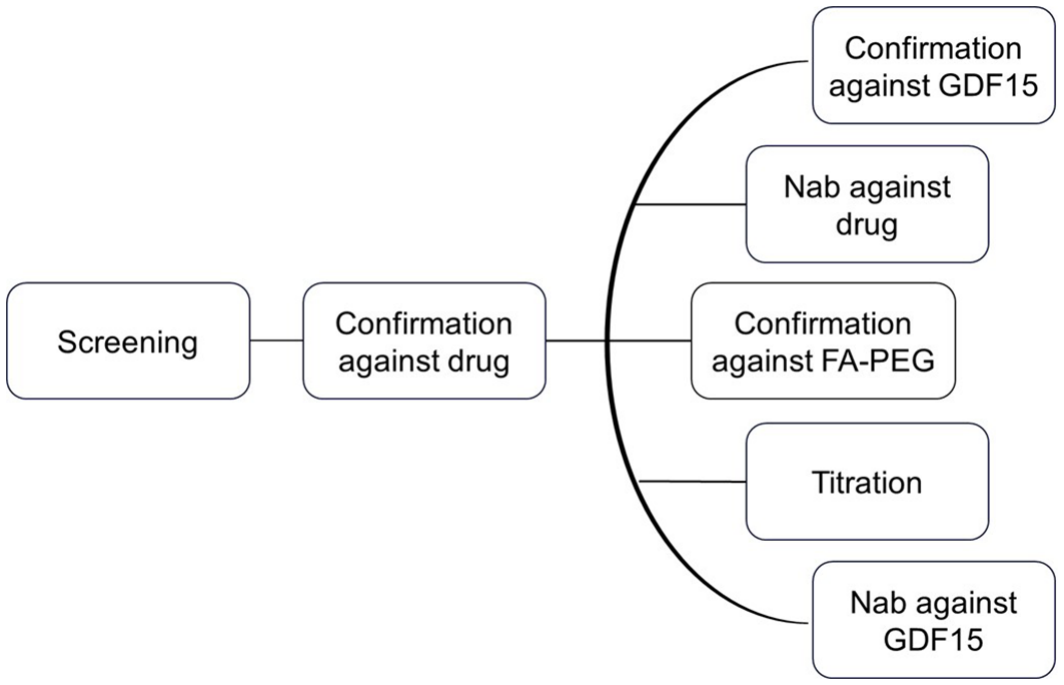
Clinical testing strategy for immunogenicity. The ADA assay initially screens for samples with a positive ADA response. To confirm the presence of ADAs, a drug-specific confirmatory assay is conducted. If the confirmatory assay is positive, a panel of parallel analyses is performed to further characterize the ADA response.

The clinical testing strategy for immunogenicity involves the use of screening and confirmatory assays to detect ADAs, followed by extensive characterization including titration, domain mapping, and assessment for neutralizing anti-drug antibodies (NAb) (**Figure 2**). This strategy is in line with the current recommendation of Health Authorities, *i.e*., EMA 2017 and FDA 2019 (14, 17). The ADA assay initially screens to identify samples that show a positive ADA response, and the screening assay detects both anti-drug IgG and IgM antibodies (14). To confirm the presence of ADAs, the drug-specific confirmatory assay is conducted. Once the drug-specific confirmatory assay yields positive results, a panel of parallel analysis is performed to characterize the ADA response further. Taking into consideration chemical modification of the therapeutic protein, characterization of the ADA response to evaluate drug domain specificity is employed. Here, a GDF15 spike to the sample leads to ADA/GDF15 complex formation and suppression of GDF15-specific ADA signals in comparison with a buffer-spiked sample. Similarly, the ADA confirmatory PEG linker + fatty acid assay focuses on the ADA response related to the PEG linker + fatty acid domain, when signals induced by PEG linker + fatty acid domain-spiked samples are compared with respective buffer-spiked sample signals. ADA titration provided semiquantitative characterization of the magnitude of the immune response in study samples.

Since the therapeutic drug is designed to mimic the endogenous counterpart (GDF15), in addition to the ADA characterization assays, the corresponding samples were analyzed using the drug and GDF15 NAb assays. The drug-specific NAb assay is designed to detect neutralizing antibodies against the drug, whereas the NAb GDF15 assay specifically targets the neutralizing antibodies against the endogenous GDF15.

A pseudo polyclonal positive control (PC) was created by combining three monoclonal antibodies in equal amounts to produce an equimolar mixture (14, 18). The pseudo-PC was then used to characterize the assays and served as a control to ensure consistent performance for ADA assays. This novel approach allowed a streamlined process of resupplying critical reagents and simplifying the assessment as well as requalification of assays of an extensive immunogenicity assessment package.

### ADA validation results

The determined screening assay cut point (SCP) factor was 1.35. The SCP data set was characterized by an inhomogeneous signal distribution with elevated signals in several drug-naive samples. Most of these high signal samples were identified as outliers in the Tukey box plot analyses and thus removed prior to SCPF calculation. Therefore, the false positive rate (FPR) on the cut point data set including outliers was 17.6% and the FPR excluding outliers was 9.6% (27). While the 9.6% is significantly larger than the targeted FPR of 5%, following a conservative screening strategy, a higher false positivity was accepted to be able to detect induced immunogenicity in low signal samples.

Multiple confirmatory cut points corresponding to the different domains were assessed. The Confirmatory cut point for the drug (CCP-I) was 29.5%. Even though in both SCP and CCP-I data sets three biological outliers were identified, these outlier samples were different for screening and confirmatory assay and none of the samples with high signals in the screening assay could be confirmed in the confirmatory assay. This suggested that the high signals in part of the SCP data set were not caused by preexisting ADAs (*e.g*., against the PEG linker + fatty acid domain). The FPR on data set including outliers was 2.0%, and the FPR excluding outliers was 0.7% which is considered acceptable for the drug confirmatory assay, where 1% FPR was targeted (27). The confirmatory cut point for GDF15 (CCP-II) was 24.8%. The FPR on the cut point data set including outliers was 1.7%, and the FPR excluding outliers was 0.6%. Considering the data set size (total n = 180), 0.6% FPR is considered acceptable for the characterizing confirmatory assays, where 1% FPR was targeted (27). The confirmatory cut point for PEG linker + fatty acid (CCP-III) was 18.0%. The FPR on the cut point data set including outliers was 2.2%, and the FPR excluding outliers was 0.6%. Considering the data set size (total n = 180), 0.6% FPR is considered acceptable for the characterizing confirmatory assays, where 1% FPR was targeted (27). The titer cut point factor (TCPF) was determined based on the screening cut point (CP) dataset. The TCPF was defined with a robust-parametric approach in the log-transformed data set with outliers removed as 2.2 (anti-log of median + 3.09*1.4826*MAD). The TCPF targets a 0.1% false positive rate (27).

The high positive control concentration level for the ADA assay was set to be at the upper third of the linear range of a surrogate antibody titration. The low positive control level was determined statistically to fail in 1% of cases as recommended by Shankar et al. (16). An intermediate LPC (LPC2) was selected as 1.5× of the 1% failure LPC level.

The developed ADA assays were validated in line with guidance document (14) evaluating parameters such as the screening assay cut point factor (SCPF), the titration assay cut point factor (TCPF), confirmatory cut points for drug (confirmatory I), GDF15 (confirmatory II) and PEG linker + fatty acid (confirmatory III), assay sensitivity and precision for screening and drug confirmatory (not for GDF15 and PEG linker + fatty acid confirmatory), assessment of the assay selectivity and specificity (interference with BMP-7), short-term stability, drug tolerance, robustness, and hook effect (see **Table 1**).

**Table 1:** ADA validation parameters for screening, confirmatory, and titration assays.

ADA assay	Cut point (% inhibition)	Sensitivity (ng/mL)	Positive control (ng/mL)	Intra (I) and inter (II) precision (CV%)	Drug tolerance (μg/mL)	Interference (hemolysis, lipemic, structurally similar compound)	Robustness
Screening	SCPF: 1.35	65.5 SPC	High PC: 10000 SPC Low PC1: 89.7 SPC Low PC2: 134.5 SPC	(I) HPC: 5.6%, LPC1: 10.9%, LPC2: 5.2% (II) HPC: 46.6%, LPC1: 18.6%, LPC2: 19.9%	Up to 4 µg/mL drug for SPC at 100 ng/mL	No interference in hemolyzed nor lipemic samples. Up to 200 pg/mL BMP-7 for SPC at LPC1, LPC2 and HPC levels	Yes
Confirmatory	CCP-I (drug): 29.5% CCP-II (GDF15): 24.8% CCP-III (PEG-fatty acid): 18.0%	67.8 SPC (drug confirmatory assay)	High PC: 10,000 SPC Low PC1: 89.7 SPC Low PC2: 134.5 SPC	Drug confirmatory assay (I) HPC: 0.2%, LPC1: 15.2%, LPC2: 3.3% (II) HPC: 1.4%, LPC1: 24.3%, LPC2: 15.3%			
Titer	TCPF: 2.2	n.a.	High PC: 10,000 SPC Low PC1: 89.7 SPC Low PC2: 134.5 SPC	n.a.			

n.a., not applicable.

### Neutralizing antibody assay validation

The ability to inhibit the activity of the drug and its endogenous counterpart GDF-15 has been addressed with the use of a cell-based assay. Downstream phosphorylation of ERK in cells expressing the human GFRAL and RET receptor served as a marker of tested protein activity after exposure to patient sera.

For the NAb assay, considering its low dynamic range, the HPC concentration level was set at the upper inhibition plateau. The low positive control level was determined statistically to fail in 1% of cases, as recommended by Shankar et al. (16). An intermediate LPC (1.5× LPC) was selected as 1.5× of the 1% failure LPC level.

The developed NAb assays were validated with respect to the following parameters: assay cut point (CP), assay sensitivity, assay precision/reproducibility, robustness, assessment of the assay selectivity (interference with drug), hemolyzed or lipemic sample, and structurally similar compound (BMP-7) (NCBI protein blast showed 33% amino acid identity)). BMP7 in human serum can interfere with MAPK signaling and ERK phosphorylation (32, 33). All validation parameters performed well within the acceptance limits required for support of clinical trials (see **Table 2**).

**Table 2:** Assay validation parameters for neutralizing anti-drug and neutralizing anti-GDF-15 assays.

Assay	Cut point (% inhibition)	Sensitivity (ng/mL)	Positive control (ng/mL)	Intra (I) and inter (II) precision (CV%)	Drug tolerance (μg/mL)	Interference (hemolysis)	Interference (lipemic)	Interference (structurally similar compound)	Robustness
Neutralizing anti-drug	38.4	2110	HPC: 10 000 1.5 × LPC1: 3730 LPC1: 2,480	I) HPC: 1.5 1.5× LPC: 2.4 LPC: 6.0 II) HPC: 1.7 1.5× LPC: 9.1 LPC: 13.0	≥ 0.427 at LPC1	No interference	No interference	No interference	Yes
Neutralizing Anti-GDF-15	28.2	1980	HPC: 10 000 1.5× LPC1: 3,220 LPC1: 2,140	I) HPC: 1.7 1.5× LPC: 4.7 LPC: 29.0 II) HPC: 2.8 1.5× LPC: 12.6 LPC: 24.6	> 0.68 at LPC1	No interference	No interference	No interference	Yes

### Evaluation of clinical immunogenicity

The described methodology for ADA assessment was implemented in two clinical trials to measure clinical samples and evaluate the immunogenicity of the drug.

In the first clinical trial, which involved a population of healthy volunteers, a single subcutaneous ascending dose of the drug was administered. None of the pre-dose samples analyzed showed positive ADA results. Out of the 47 subjects, emergent ADAs against the drug were confirmed in only two individuals. The two ADA-positive individuals showed scores just above the confirmatory cutoff point (CP) in the drug confirmatory assay. Upon GDF15 and PEG-fatty acid characterization of the response, the two ADA confirmatory characterization assays could not confirm the positive results from the drug confirmatory assay. Also, a titer was not detectable at 1:2 dilution in both samples (assuming prior MRD 50). For one of the subjects, later timepoints assessed scored negative in the ADA assay, indicating a transient nature of ADA response. For the second subject, the ADA-positive sample was detected at the end of a study visit, therefore indicating a persistent nature of a response. Furthermore, no neutralizing capacity of the ADA was detected for the drug when performing a cell-based NAb assay analysis.

In the second clinical study, which involved an overweight or obese population, the drug was administered subcutaneously with different dosing regimens. ADAs against the drug were confirmed in 3 of the 82 patients. One individual showed a confirmed positive ADA result in the pre-dose sample, whereas two individuals had emergent confirmed positive ADA results after the dose administration. Similar to the outcome in the first clinical trial, domain characterization for PEG-fatty acid showed a negative result. However, the characterizing ADA confirmatory assay for GDF15 confirmed the two positive results obtained from the drug confirmatory assay. Further characterization for the neutralizing capacities of ADA revealed positive data in the NAb assay for GDF15, but not for the drug itself. The NAb GDF15 results for the three ADA-confirmed positive samples were all in the range between CP and LPC, suggesting low neutralizing activity. Interestingly, the drug NAb assay detected those samples as negative. Their inhibition level in the drug NAb assay was elevated but below the CP. The different outcome in scoring above CP (NAb GDF15) and below CP (NAb whole drug) can be attributed to the subtle differences in assay sensitivity between one NAb assay format vs. the other and run-to-run variability, which also was observed during assay validation. No detectable titer was observed at a 1:2 dilution in all samples, assuming a prior MRD of 50. In each case, ADAs were detected in the patients only once throughout the studies, indicating the transient nature of their appearance (see **Table 3**). In both clinical trials, the observed incidents of ADA did not have any impact on drug exposure.

**Table 3:** Characterization of immunogenicity from two clinical trials.

Clinical study	Confirmed ADA patients Cross-reactive to the drug	Cross-reactive to FA PEG	Cross-reactive to GDF15	NAb for drug	NAb for GDF15	Titer
Healthy volunteers, 47 dosed and 64 enrolled	2 out of 47 1 transient 1 persistent	NO	NO	NO	NO	Low
Disease population, 82 dosed	3 out of 82 1 preexisting 2 transient	NO	YES 2 out of 3	NO	YES 3 out of 3	Low

## Discussion

Described in the current case study, the proposed strategy of conventical ADA detection and extensive ADA response characterization proved effective for monitoring the presence of ADAs and NAbs and could be used to support clinical development of a broad range of chemically modified proteins and multidomain biotherapeutics.

The industry advocates a risk-based approach considering drug exposure, efficacy, and patient safety when developing an immunogenicity strategy. The immune response to chemically modified endogenous molecules can be directed either to multiple epitopes across the non-modified biologics or to the chemically modified parts. For this molecule, one of the domains is a GDF15 protein with an endogenous counterpart, which is conjugated with a PEG linker coupled to a fatty acid. Since an immune response to the GDF15 domain could potentially have an impact on the regulation of multiple physiological functions, it was essential to investigate and characterize the neutralizing capacities of potential ADAs. Therefore, this novel chemically modified endogenous molecule was assessed as a high-risk biologic from the immunogenicity risk standpoint, warranting development of two neutralizing assays, one targeting the entire multidomain therapeutic and a second neutralizing assay for the GDF15 domain only. This risk assessment was reflected in the request from regulatory authorities to perform the described characterization.

A strategy of creating a pseudo polyclonal positive control was adopted during the development and validation of ADA assays. This involved the combination of three different monoclonal antibodies, derived specifically against the human GDF15, in equimolar ratios. By adopting this approach, a reliable and reproducible source of a critical reagent was established (34). This pseudo polyclonal positive control played a pivotal role in consistently evaluating the performance of ADA assays throughout the entire clinical development process, in our case consisting of two clinical studies. Moreover, the establishment of pseudo positive control that specifically binds to variable regions of the drug aligns with Health Authority guidelines (14). This original approach ensured that the control maximally represented the interaction between the drug and potential anti-drug antibodies and is highly recommended for bioanalytical community to further evaluate.

During the clinical study in a healthy volunteer population, 2 out 47 dosed individuals were confirmed ADA positive, but no neutralizing antibodies were detected. In the clinical study with an overweight or obese population, 3 of 82 patients were confirmed positive. While the observed incidence of ADA was low, through the characterization assessments performed, we were able to establish that the ADAs were cross-reactive against GDF15 and most importantly that these ADA were able to neutralize the function of GDF15, albeit at a very low titer. These observations highlight the advantage of a parallel and unbiased ADA characterization process in high-risk modalities and justify the investment in the extensive suite of assays for development of this high-risk therapeutic.

It has been reported in literature that protein conjugation with PEG may trigger an immune response and the developed anti-PEG antibodies can impact the safety and efficacy of the administered drug (10, 13). When administering therapeutics using PEG liposomes, anti-PEG IgM antibodies were detected, which could trigger the complement system, leading to accelerated blood clearance and reduced exposure and efficacy due to anti-PEG antibodies. Since this was the first time the current biotherapeutics with this PEG linker was introduced into clinical development, it was of high importance to gain understanding of potential ADA response and develop an assay which would allow the characterization of potential anti-PEG antibodies. However, for the fatty acid domain within the chemically modified therapeutic protein, the requirement to develop an assay for the characterization of the immune response was questionable. To our knowledge, anti-fatty acid antibodies have not been associated with loss of efficacy or safety concerns; therefore, this domain was not included by itself in the characterization using bioanalytical methodology (35) but rather in combination with the applied PEG linker.

No ADAs against PEG linker–fatty acid conjugates were detected while characterizing detected ADAs in two clinical studies for the current biotherapeutic. One explanation could be that preexisting ADAs against PEG known for their high prevalence would not cross-react to the PEG linker as the linker is relatively short in comparison with a PEGylated domain. It is also possible that the frequency was too low to be observed in the participants treated with the respective drug. Based on current analysis, the PEG linker can be considered suitable for conjugation of other molecules as a domain with low immunogenic potential.

Each clinical development program administrating a multidomain biotherapeutic should consider whether each domain or component requires its own specificity assay and the extent of ADA characterization to be performed. In addition to the different functional domains, it must also be considered that inter-domain interfaces created within each chemically modified biotherapeutic can themselves also trigger an immune response (34, 36, 37). Including domain characterization as part of a validated ADA assay as described in the current strategy can be analytically and operationally challenging, requiring specific critical reagents and considerable scientific effort. Although the described and successfully implemented strategy allowed us to assess and understand the clinical immunogenicity of the current drug, we propose several reflections when considering the ADA detection and characterization for multidomain biotherapeutics.

The risk assessment of the therapeutic molecule will be the major factor when deciding the extent of the characterization required. With a lower-risk molecule, a more exploratory approach to the domain specificity assessment may be sufficient in the early stages of clinical development and an assessment of the neutralizing potential may be deferred until later clinical phases. In case one of the domains has a particular impact on the ADA formation, this can support either further clinical development of respective biotherapeutics or back-translational efforts. Even within a high-risk molecule, there are likely to be domains with lower and higher risks of clinical consequences for immunogenicity. Here for example, we considered that the fatty acid domain did not require its own domain specificity assay, whereas the GDF15 domain had both ADA specificity and NAb assays.

To assess the pharmacological neutralization of a chemically modified endogenous molecule, either a cell-based or a ligand-binding assay can used to assess the capacity of the ADA to reduce the multidomain biotherapeutics potency by blocking the target binding domain. For high-risk molecules, a functional cell-based assay is generally regarded as more appropriate to characterize a potential neutralizing effect already at the Phase I entry into the human stage of clinical development since they are considered to better represent the physiological mode of action (19). Following our safety-driven conservative immunogenicity monitoring approach, in this program a cellular assay had already been developed to support the early characterization at Phase I. The engineered HEK-cell line expressing the target receptor complex hGFRAL/hRET has been utilized to assess the NAb capacity of anti-drug and anti-GDF15 antibodies.

During NAb assay development, known analytical challenges of a cell-based system, *i.e.*, sensitivity and drug tolerance compared with the corresponding ADA screening ligand-binding assay, were faced. To overcome the limitation, extensive sample preparation to break apart NAb/drug complexes and remove interfering drug and other interfering matrix components from the sample were introduced (38–40). The poor solubility of the drug prevented standard approaches for chemical conjugation with biotin that would have allowed a SPEAD pretreatment approach. Alternative sample pretreatment that did not require any chemical conjugation had to be envisioned. PEG precipitation combined with acid treatment and anti-drug antibody-mediated drug capture was therefore implemented that 1) removed interfering matrix components that would trigger unspecific ERK phosphorylation in the cellular assay and 2) removed some but not all of the drugs present in clinical samples, thereby improving the drug tolerance of the assay.

Thanks to the multiple mitigation strategies described here, we were able to achieve successful analytical validation of two NAb assays. Our methodology allowed detection of neutralizing antibodies at the expected drug concentration in the clinical studies. While performing clinical sample analysis in healthy and overweight or obese populations, we could also confirm the presence of neutralizing antibodies in three samples. Interestingly, all the samples showed a positive result only in the assay addressing the impact on GDF-15 but not on the drug itself. These unexpected results can be explained by a small difference in activity between the drug and recombinant GDF-15, which may impact the assay sensitivity at the tested conditions. However, this small distinction may lead to a different outcome if the signal inhibition is at the detection threshold.

Considering the analytical challenges related to sensitivity of the cellular NAb assays even after implementing sample pretreatment, which have been also demonstrated in the current case study, it is advised to plan for NAb assessments only at later sampling timepoints and particularly in the washout phase in studies when multiple ascending doses are explored (34). To streamline analytics development and characterization of ADAs, one could develop only one NAb assay to assess neutralizing ADAs against the endogenous counterpart, but not against the whole drug itself, which would still address the major safety concerns. Additionally, a non-cell-based competitive ligand-binding assay-based NAb assay might allow better analytical performance to assess neutralizing ADA, particularly with respect to sensitivity and drug tolerance. One could consider implementation of competitive ligand-binding assay NAb assay at the earlier stages of clinical development instead of a cell-based assay. Depending on the mechanism of action of the therapeutic, such an approach may be appropriate to support the entire clinical development (40).

For a complex multidomain therapeutic, particularly one with higher-risk components, it is recommended that the strategy for ADA detection and characterization, including the approach for neutralizing ADA assessment, be discussed with Health Authorities prior to the Investigational New Drug (IND) applications stage of drug development.

## Data Availability

The original contributions presented in the study are included in the article/supplementary material. Further inquiries can be directed to the corresponding author.
